# Irreversible electroporation combined with chemotherapy and PD-1/PD-L1 blockade enhanced antitumor immunity for locally advanced pancreatic cancer

**DOI:** 10.3389/fimmu.2023.1193040

**Published:** 2023-08-25

**Authors:** Yangyang Ma, Yanli Xing, Hongmei Li, Ting Yuan, Bing Liang, Rongrong Li, Jianyu Li, Zhonghai Li, Shuying Li, Lizhi Niu

**Affiliations:** ^1^Central Laboratory, Affiliated Fuda Cancer Hospital, Jinan University, Guangzhou, China; ^2^Department of Oncology, Affiliated Fuda Cancer Hospital, Jinan University, Guangzhou, China; ^3^Department of Surgery and Anesthesia, Affiliated Fuda Cancer Hospital, Jinan University, Guangzhou, China; ^4^Department of Ultrasound, Affiliated Fuda Cancer Hospital, Jinan University, Guangzhou, China; ^5^Department of Radiology, Affiliated Fuda Cancer Hospital, Jinan University, Guangzhou, China

**Keywords:** irreversible electroporation, locally advanced pancreatic cancer, chemotherapy, PD-1/PD-L1 blockade, anticancer immunity

## Abstract

**Background:**

Irreversible electroporation (IRE) is a novel local tumor ablation approach with the potential to stimulate an antitumor immune response. However, it is not effective in preventing distant metastasis in isolation. This study aimed to compare the potential of augmenting the antitumor immune response in patients with locally advanced pancreatic cancer (LAPC) who underwent IRE combined with chemotherapy and PD-1/PD-L1 blockade with those who underwent IRE combined with chemotherapy.

**Methods:**

A retrospective review was conducted on LAPC patients treated either with IRE in combination with chemotherapy and PD-1/PD-L1 blockade (group A) or with IRE with chemotherapy alone (group B) from July 2015 to June 2021. The primary outcomes were overall survival (OS) and progression-free survival (PFS), with immune responses and adverse events serving as secondary endpoints. Risk factors for OS and PFS were identified using univariate and multivariate analyses.

**Results:**

A total of 103 patients were included in the final analysis, comprising 25 in group A and 78 in group B. The median duration of follow-up was 18.2 months (3.0–38.6 months). Group A patients demonstrated improved survival compared to group B (median OS: 23.6 *vs*. 19.4 months, *p* = 0.001; median PFS: 18.2 *vs*. 14.7 months, *p* = 0.022). The data suggest a robust immune response in group A, while adverse events related to the treatment were similar in both groups. The multivariate analysis identified the combination of IRE, chemotherapy, and PD-1/PD-L1 blockade as an independent prognostic factor for OS and PFS.

**Conclusion:**

The addition of PD-1/PD-L1 blockade to the regimen of IRE combined with chemotherapy enhanced antitumor immunity and extended survival in LAPC patients.

## Introduction

Pancreatic cancer represents a particularly aggressive form of malignancy accompanied by a discouragingly poor prognosis. The 5-year survival rate for patients diagnosed with pancreatic cancer remains under 8% (1). While surgical resection is currently the only treatment offering a potential cure, most patients present with locally advanced pancreatic cancer (LAPC) at diagnosis, and only less than 20% of newly diagnosed patients are eligible for surgical resection (2, 3). Furthermore, LAPC exhibits low responsiveness to conventional chemoradiotherapy, resulting in only marginal improvement in survival (4).

Conventional thermal local ablation techniques, such as radiofrequency, microwave, and cryoablation, are currently employed to manage patients with LAPC (5–9). However, due to the heat sink effects, these thermal ablation methods may damage peripancreatic vessels, the duodenum, and the bile and pancreatic duct, leading to high morbidity and mortality (10, 11). A potential solution to this challenge is irreversible electroporation (IRE). This innovative, nonthermal local ablation technique leverages high-voltage electrical pulses to disrupt cell membranes, resulting in irreversible nanoscale perforations and subsequent apoptotic cell death (12, 13). Compared to its thermal counterparts, IRE offers the advantage of sparing essential structures such as blood vessels, bile ducts, and nerves since the procedure generally unaffected extracellular matrix and collagen structures (14, 15).

Our prior research has also indicated that the antitumor effectiveness is further amplified when IRE is used concurrently with chemotherapy. This synergistic effect is attributed to IRE’s ability to enhance cell membrane permeability, thereby facilitating superior drug diffusion into the cells and boosting cytotoxicity (16, 17). This combination therapy presents an enticing treatment option for LAPC patients.

Immune checkpoint inhibitors (ICIs), which modulate immune responses against tumors, have substantially transformed therapeutic strategies in oncology. Several hypermutated cancers, including melanoma and non-small cell lung cancer, have shown responsiveness to ICIs due to the production of neoantigens and the presence of abundant tumor-infiltrating lymphocytes, thus positioning ICIs as a front-line treatment for these conditions (18–20). However, patients with LAPC typically exhibit a lower response rate to ICIs, attributed to reduced immunogenicity, fewer neoantigens, and lower immune infiltration (21, 22).

Emerging evidence suggests that IRE can facilitate T-cell immunity and activate tumor antigen-specific CD8^+^ T cells (23–25). IRE not only causes minimal damage to blood vessels, but also enhances vascular permeability, leading to rapid transportation of CD8^+^ T lymphocytes to the tumor core and subsequent activation of the immune system (23, 26). However, clinical studies indicate that IRE alone can only transiently mitigate the immunosuppressive state by decreasing the frequency of regulatory T cells (Tregs) (23, 27). IRE alone is insufficient in halting cancer progression.

Given these findings, combining IRE with chemotherapy and ICIs might present a promising strategy with the potential to elicit a powerful and enduring antitumor immune response in LAPC patients. There has been limited focus on this aspect, necessitating our current study that seeks to evaluate the antitumor immunity in LAPC patients following the combined application of IRE, chemotherapy, and ICIs.

## Materials and methods

### Patients

This retrospective study was approved by the Institutional Ethic Committee of Fuda Cancer Hospital and conducted in accordance with the Declaration of Helsinki and the Declaration of Good Clinical Practice. Written informed consent was obtained from each participant. From July 2015 to June 2021, all patients with LAPC underwent either a combination of IRE with chemotherapy and PD-1/L1 blockade (group A) or a combination of IRE with chemotherapy (group B). The inclusion criteria were as follows: (1) histologically confirmed pancreatic adenocarcinoma and radiologically verified LAPC [LAPC was defined in the 8th edition of the American Joint Committee on Cancer (AJCC) staging system for pancreatic cancer] (28); (2) patients over 18 years; (3) sufficient bone marrow, liver, kidney, and coagulation function; and (4) performance status (PS) scores under 2. The exclusion criteria were as follows: (1) patients with distant metastases; (2) patients who underwent other forms of treatment; and (3) patients with incomplete or missing data.

### Treatment procedure

Before the IRE procedure, patients received 1,000 mg/m^2^ of gemcitabine intravenously (over 30 min). During the IRE, all patients were placed in a supine position under general anesthesia. Non-depolarizing neuromuscular blocking agents (vecuronium bromide and rocuronium bromide) were administered to reduce muscle contraction. A CT scanner and ultrasound system were used to confirm the morphology and surrounding relationships of the tumor. The number of electrode needles (2–6) and the approach route were determined according to tumor size and location. The exposed length of the probe tip was approximately 10–20 mm, and the needle distance was 15–25 mm. The ablation parameters were as follows: voltage 1,500 V/cm, number of pulses 90–100, and pulse width 70–90 μs. After releasing a group of pulses, the current rise was adjusted at 12–15 A, and the maximum was close to 50 A. For larger diameter tumors, ablation was repeated after retreating the needle by 1 cm until the ablation area covered the whole lesion. A CT scan or ultrasonography of the abdomen was performed again after the ablation to determine whether the ablation had been completed, whether bleeding had occurred, and whether essential structures had been damaged. When the disease was stable, oral S1 or albumin combined with paclitaxel was administered as maintenance therapy.

**Figure 1:**
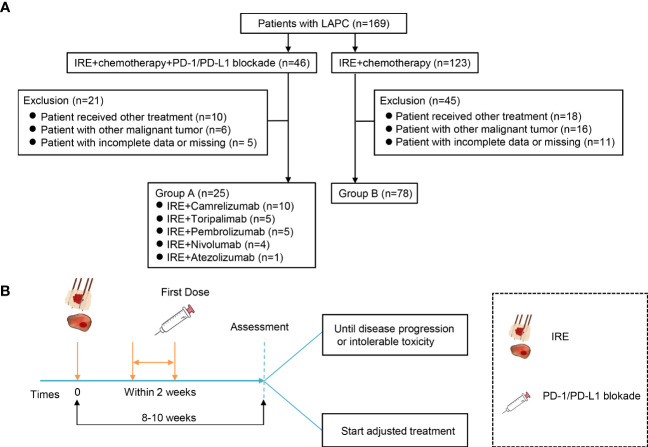
Flowchart of the included patients and treatment protocol. **(A)** Flowchart of the included patients. A total of 169 patients with LAPC were included, and 66 participants were excluded. Finally, 25 patients were analyzed in group A and 78 in group B. **(B)** Treatment protocol. Group A treated with a combination of IRE with chemotherapy and PD-1/PD-L1 blockade. Patients received PD-1/PD-L1 blockade every 2/3 weeks for four doses until disease progression or intolerable toxicity after IRE, and group B was treated with combination of IRE with chemotherapy.

Patients in group A received PD-1/PD-L1 blockade therapy, including camrelizumab (200 mg/2 weeks), toripalimab (240 mg/2 weeks), nivolumab (100 mg/2 weeks), pembrolizumab (100 mg/3 weeks), and atezolizumab (1,200 mg/3 weeks). The first dose of PD-1/PD-L1 blockade was administered within 2 weeks after IRE. Patients received continuous PD-1/PD-L1 blockade treatment until intolerance toxicity or progressive disease. The treatment protocol is illustrated in **Figure 1B**.

### Assays of immune parameters

Blood samples of each patient’s blood (2 ml) were collected just before the IRE procedure (Pre-IRE) and 30 days (Post-IRE) after treatment. Flow cytometry techniques (FACS caliber, four-color system, BD Bioscience, CA, US) were used to analyze the absolute number of CD3^+^ T cells (CD3^+^), CD4^+^ T cells (CD3^+^CD4^+^), CD8^+^ T cells (CD3^+^CD8^+^), NK cells (CD16^+^CD56^+^), T-lymphocytes PD-1 (CD3^+^PD-1^+^), T helper lymphocytes PD-1 (CD4^+^PD-1^+^), cytotoxic T lymphocytes PD-1 (CD8^+^PD-1^+^), and Treg cells (CD4^+^CD25^+^CD127dim). A similar method was used to measure serum cytokines, including interleukin-6 (IL-6), interleukin-10 (IL-10), tumor-necrosis factor-beta (TNF-β), and interferon-γ (IFN-γ). A record of the data was then made.

### Collection of clinical data

The clinical information and demographic were collected, including age, gender, lesion size, tumor location, performance status (PS), carbohydrate antigen 19-9 (CA19-9), preoperative therapy, PD-L1 expression, and PD-1/L1 blockade. The tumor response was evaluated based on the mRECIST (29). The objective response rate (ORR) was calculated as follows: ORR = (CR+PR cases)/total cases × 100%. OS was defined as the time from diagnosis to death or until the last follow-up. PFS was defined as the time from diagnosis to disease progression until the last follow-up.

### Follow-up

All patients in this study were followed up at 1, 3, and 6 months post-IRE and then every 3 months. Two radiologists independently interpreted all follow-up scan results. Adverse events were evaluated and classified according to the National Cancer Institute Common Terminology Criteria for Adverse Events, version 4.0. The last follow-up was completed on 25 February 2022.

### Statistical analysis

All statistical analyses were performed and compiled using GraphPad Prism (version 8.01, GraphPad Software, San Diego, CA, USA) and SPSS software (version 20.0, SPSS Inc., Chicago, IL, USA). Categorical variables were compared using the chi-square or Fisher exact test. The continuous data, categorical data, and survival curves of the two groups were compared using the Mann–Whitney test, Fisher’s exact test, and log-rank (Mantel-Cox) tests individually. The Cox regression model was used in survival analysis to assess the influence factor of OS and PFS. All statistical tests were two-sided; a *p*-value < 0.05 was considered statistically significant.

## Results

### Patient characteristics

Between July 2015 and June 2021, a total of 169 LAPC patients were included in this study (**Figure 1**). Based on the exclusion criteria, 66 patients were excluded owing to receiving other treatment (*n* = 28), tumor metastasis (*n* = 22), incomplete data, or missing (*n* = 16). Finally, a total of 103 LAPC patients were analyzed in this study: group A (*n* = 25) and group B (*n* = 78) (**Figure 1A**). The clinical characteristics of the patients were well-balanced between the two groups (**Table 1**). The median ages were 55 (26-80) and 62 (34-78) years in groups A and B, respectively. Both groups had a higher proportion of male patients than female patients. The median tumor sizes were 4.1 cm (range 2.4–6.7) and 3.8 cm (range 2.1–6.2) for patients in groups A and B, respectively. Group A had a higher tumor burden, but the difference was not statistically significant.

**Table 1:** Patient characteristics.

Characteristic	Group A (*n* = 25)	Group B (*n* = 78)	*p-*value
Age, years			0.308
≤60	16	51	
>60	9	17	
Sex			
Female	17	55	0.807
Male	8	23	
Tumor size (cm)			0.337
2–4	14	53	
≥4	11	25	
Tumor location			0.205
Head	19	60	
Body/Tail	6	18	
PS			0.645
0	4	14	
1	15	38	
2	6	26	
CA 19-9 (U/ml)			0.812
≤37	8	28	
>37	17	50	
Preoperative therapy			0.829
Hepaticojejunostomy	15	48	
Gastrojejunostomy	7	24	
Cholecystectomy	3	6	
Chemotherapy before IRE			0.731
Gemcitabine	7	18	
S-1	9	25	
FOLFIRINOX	9	35	
PD-L1 expression, *n* (%)			0.412
Positive	6	30	
Negative	2	7	
Unknown	17	41	
PD-1/L1 blockade, *n* (%)			
Camrelizumab	10	0	
Toripalimab	5	0	
Nivolumab	4	0	
Pembrolizumab	5	0	
Atezolizumab	1	0	

IRE, irreversible electroporation; PD-1, programmed cell death protein 1; PD-L1, programmed death-ligand 1; PS, performance status; CA19-9, carbohydrate antigen 19-9.

### Adverse events

As shown in **Table 2**, there was no treatment-related mortality in either group in our study. The overall adverse events rate was similar between the two groups. The most common immune-related adverse events were pruritus (24%), hypothyroidism (16%), increased bilirubin (16%), and ALT increase (16%) in group A. The major (grade 3–4) immune-related adverse events were hypothyroidism (8%), ALT increased (4%), and colitis (4%) in group A, which was resolved after the treatment with a corticosteroid. There were no immune-related adverse events in group B. The most common IRE treatment-related adverse events were pain (72%), fatigue (64%), diarrhea cardiac (56%), arrhythmias (48%), and hypertension (48%) in group A. The major (grade 3–4) IRE-related adverse events were cardiac arrhythmias (48%), hypertension (48%), pancreatitis (28%), and hemorrhage (16%). All adverse events were relieved or improved after symptomatic treatment.

**Table 2:** Rates of the adverse events after treatment [*n* (%)].

Adverse event	Group A (*n* = 25)		Group B (*n* = 78)	
	All Grade	Grade 3/4	All Grade	Grade 3/4
Immune related				
Pruritus	6 (24.0)	0	0	0
Hypothyroidism	4 (16.0)	2 (8)	0	0
Bilirubin increased	4 (16.0)	0	0	0
ALT increased	4 (16.0)	1 (4)	0	0
Colitis	3 (12.0)	1 (4)	0	0
Diarrhea	2 (8)	0	0	0
Cough	2 (8)	0	0	0
Fever	1 (4)	0	0	0
Autoimmune disorder	1 (4)	0	0	0
Adrenal insufficiency	1 (4)	0	0	0
Fatigue	1 (4)	0	0	0
Oral pain	1 (4)	0	0	0
WBC decreased	1 (4)	0	0	0
IRE treatment related				
Pain	18 (72)	0	62 (79.4)	0
Fatigue	16 (64)	0	45 (57.7)	0
Diarrhea	14 (56)	0	36 (46.2)	0
Cardiac arrhythmias	12 (48)	2 (8)	26 (33.3)	7 (8.9)
Hypertension	12 (48)	3 (12)	36 (46.2)	9(11.5)
Pancreatitis	7 (28)	1 (4)	22 (28.2)	4 (5.1)
Hemorrhage	4 (16)	1 (4)	12 (15.3)	2 (2.6)
Biliary fistula	6 (24)	0	17 (21.8)	3 (3.8)
Nausea and vomiting	5 (20)	0	14 (17.9)	0
Infection	5 (20)	0	15 (19.2)	0
Fever	5 (20)	0	13 (16.6)	0
Loss of appetite	3 (12)	0	8 (10.2)	0
Ascites	3 (12)	0	9 (11.5)	0
Pleural effusion	2 (8)	0	4 (5.1)	0
Abdominal distention	1 (4)	0	5 (6.4)	0

### Response

**Figure 2:**
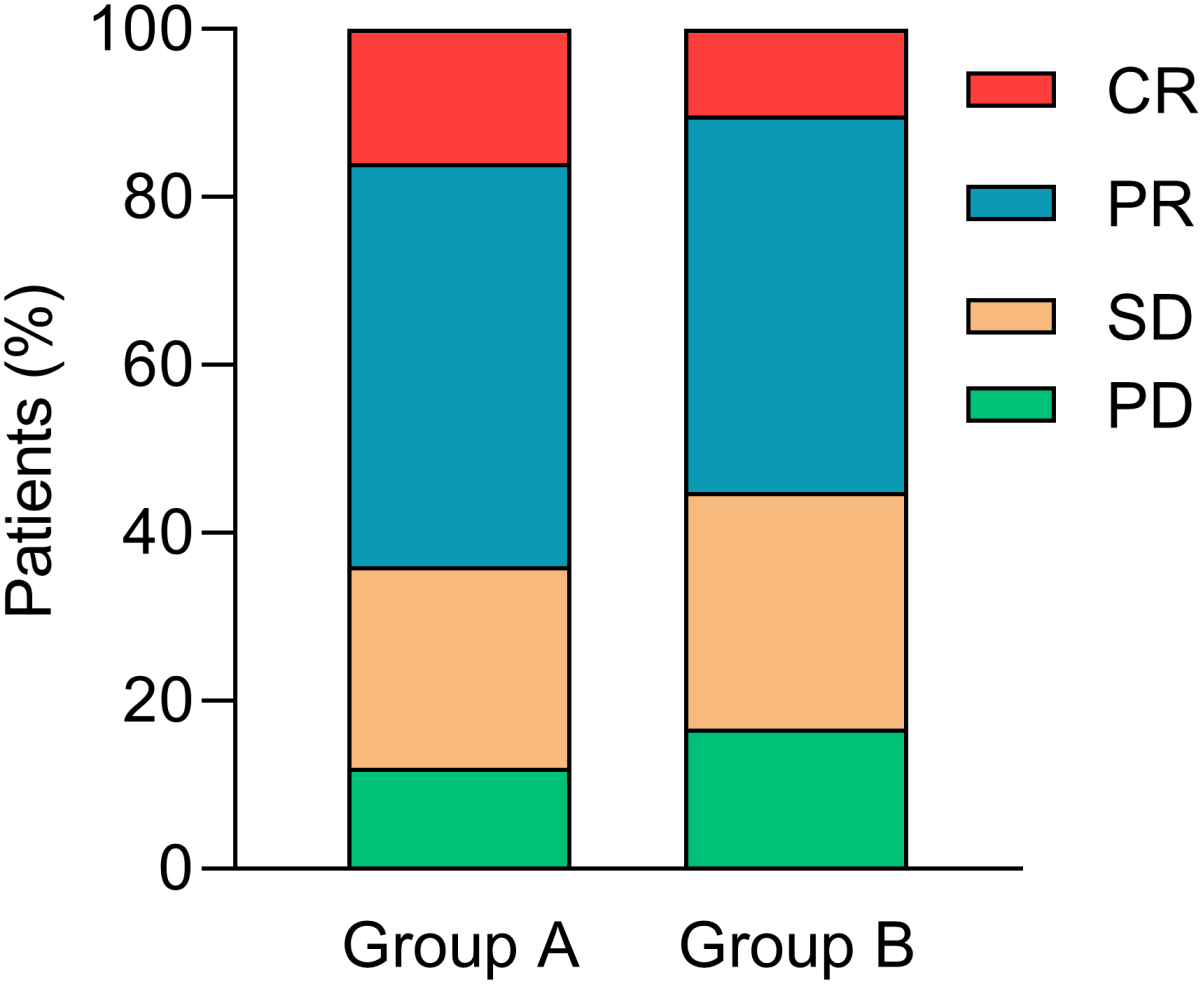
Tumor responses. CR, complete response; PR, partial response; SD, stable disease; PD, disease progression.

Overall, 4 (16.0%) patients achieved CR, 12 (48.0%) patients received PR, 6 (24.0%) patients developed SD, and 3 (12.0%) patients suffered PD in group A. The tumor response in group B was 8 (10.3%) patients with CR, 35 (44.8%) patients with PR, 22 (28.2%) patients with SD, and 13 (16.7%) with PD. The ORR was 64.0% *vs*. 55.1% (*p* = 0.787, **Figure 2**) in groups A and B, respectively.

### Immune parameters

**Figure 3:**
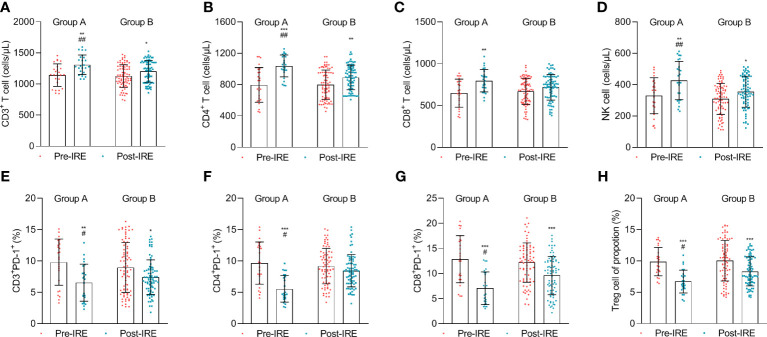
The number of lymphocytes in the blood was measured before treatment (Pre-IRE) and after 30 days (Post-IRE) of treatment. **(A)** The number of CD3^+^ T cells, **(B)** CD4^+^ T cells, **(C)** CD8^+^ T cells, **(D)** NK cells, **(E)** T-lymphocytes PD-1, **(F)** T helper lymphocytes PD-1 (CD4^+^ PD-1^+^), **(G)** cytotoxic T lymphocytes PD-1, and **(H)** Tregs cells were analyzed by multicolor flow cytometry. Comparison within groups: *p < 0.05; **p < 0.01; ***p < 0.001; comparison between groups: #*p* < 0.05; ##*p* < 0.01.

We compared the absolute number of immune cells after combined IRE, chemotherapy, and PD-1/PD-L1 blockade to investigate post-treatment immune effects. The results demonstrated that the absolute number of CD3^+^ T cells, CD4^+^ T cells (*p* = 0.038), CD8^+^ T cells (*p* = 0.024), and NK cells steadily increased (**Figures 3A–D**), while the proportion of CD3^+^PD-1^+^, CD4^+^PD-1^+^, CD8^+^PD-1^+^, and Tregs cells (*p* = 0.023) decreased after treatment in group A compared to group B (**Figures 3E–H**).

**Figure 4:**
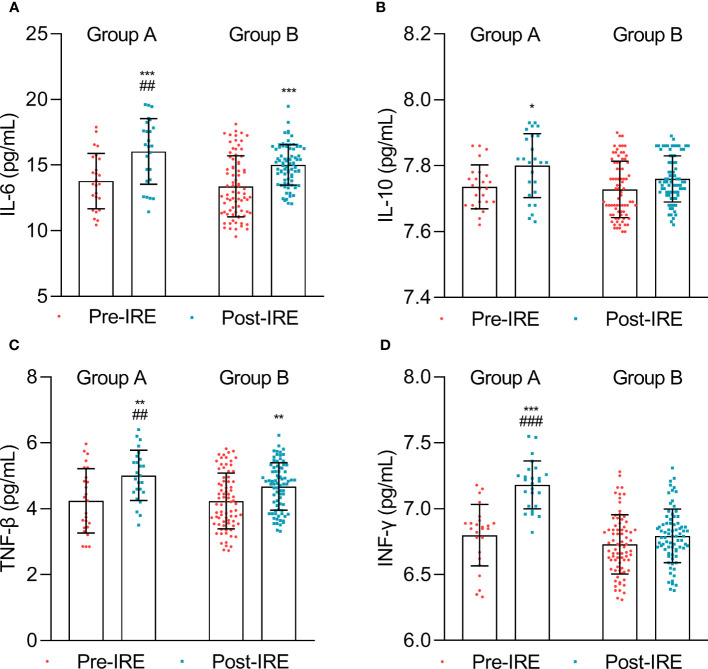
Cytokine expression measured before treatment (Pre-IRE) and after 30 days (Post-IRE). **(A)** The numbers of IL-6, **(B)** IL-10, **(C)** TNF-β, and **(D)** INF-γ were analyzed by multicolor flow cytometry. Comparison within groups: *p < 0.05; **p < 0.01; ***p < 0.001; comparison between groups: ##*p* < 0.01; ###*p* < 0.001.

To further compare the variations of the immune response, we compared the levels of cytokines in both groups. The data suggested that the IL-6, IL-10, TNF-β, and IFN-γ levels significantly increased after treatment in group A, while the change in IL-10 and IFN-γ was not significant after treatment in group B (**Figure 4**).

### Survival

**Figure 5:**
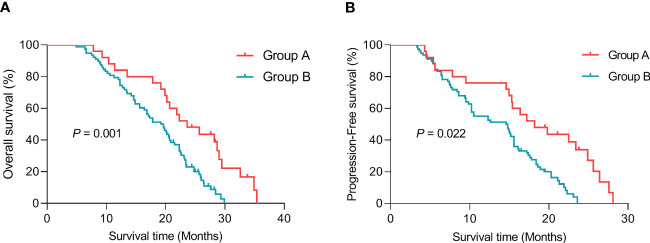
Graph showing Kaplan–Meier survival curves. **(A)** Median overall survival (OS) from diagnosis. **(B)** Median progression-free survival (PFS) from diagnosis.

The median follow-up time was 18.2 months (3.0–38.6 months). The median OS from diagnosis was significantly longer in group A than in group B, respectively (23.6 months *vs*. 19.4 months, *p* = 0.001, **Figure 5A**). Similarly, the median PFS from diagnosis was significantly longer in group A than in group B, respectively (18.2 months *vs*. 14.7 months, *p* = 0.022, **Figure 5B**).

### Univariate and multivariate analyses of OS and PFS

As shown in **Table 3**, tumor size (HR: 1.022, 95% CI: 1.001–1.043, *p* = 0.045), CA 19-9 (HR: 1.021, 95% CI: 0.095–1.024, *p* = 0.017), and IRE plus PD-1/PD-L1 blockade treatment (HR: 2.217, 95% CI: 1.161–5.034, *p* = 0.018) were significantly correlated with OS in the univariate Cox regression analysis. Multivariate analysis showed that the combination of IRE with chemotherapy and PD-1/PD-L1 blockade (HR: 3.605, 95%, 95% CI: 1.417–9.175, *p* = 0.007) were independent predictors of OS (**Table 3**). Furthermore, tumor number (HR: 1.756, 95% CI: 0.956–1.327, *p* = 0.024) and IRE plus PD-1/PD-L1 blockade treatment (HR: 0.459, 95% CI: 0.248–0.824, *p* = 0.006) were also significant prognostic factors for PFS (**Table 4**).

**Table 3:** Univariate and multivariate analyses of OS in patients.

Characteristic	Group A (*n* = 25)		Group B (*n* = 78)	
	Univariate analysis		Multivariate analysis	
	HR (95% CI)	*p*-value	HR (95% CI)	*p*-value
Age, years				
≤60	Reference		Reference	
>60	0.756 (0.238–0.968)	1.263		
Sex				
Female	Reference		Reference	
Male	0.366 (0.210–0.637)	0.568		
Tumor size (cm)				
2–4	Reference		Reference	
≥4	1.022 (1.001–1.043)	0.045	1.002 (0.951–1.055)	0.949
Tumor location				
Head	Reference		Reference	
Body/Tail	0.656 (0.321–1.575)	0.385		
PS				
0	Reference		Reference	
1	1.004 (0.998–1.01)	0.213		
2	0.956 (0.453–2.085)	0.941		
CA 19-9 (U/ml)				
≤37	Reference		Reference	
>37	1.021 (0.095–1.024)	0.017	0.361 (0.142–1.015)	0.062
Preoperative therapy				
Hepaticojejunostomy	Reference		Reference	
Gastrojejunostomy	0.925 (0.625–1.417)	0.768		
Cholecystectomy	1.125 (0.476–2.718)	0.754		
Chemotherapy before IRE				
Gemcitabine	Reference		Reference	
S-1	0.685 (0.308–1.527)	0.355		
FOLFIRINOX	0.623 (0.256–1.424)	0.256		
PD-L1 expression, *n* (%)				
Positive	Reference		Reference	
Negative	0.622 (0.309–1.435)	0.224		
Unknown	0.826 (0.456–1.485)	0.525		
PD-1/L1 blockade, *n* (%)				
Camrelizumab	Reference		Reference	
Toripalimab	0.685 (0.389–1.225)	0.225		
Nivolumab	0.656 (0.245–1.428)	0.236		
Pembrolizumab	0.625 (0.378–1.489)	0.356		
Atezolizumab	1.465 (0.835–2.559)	0.112		
Treatment				
IRE+chemotherapy	Reference		Reference	
IRE+chemotherapy+PD-1/PD-L1 blockade	2.217 (1.161–5.034)	0.018	3.605 (1.417–9.175)	0.007

IRE, irreversible electroporation; OS, overall survival; PD-1, programmed cell death protein 1; PD-L1, programmed death-ligand 1; HR, hazard ratio; CI, confidence interval; CA19-9, carbohydrate antigen 19-9.

**Table 4:** Univariate and multivariate analyses of PFS in patients.

Characteristic	Group A (*n* = 25)		Group B (*n* = 78)	
	Univariate analysis		Multivariate analysis	
	HR (95% CI)	*p*-value	HR (95% CI)	*p*-value
Age, years				
≤60	Reference		Reference	
>60	1.189 (0.674–2.032)	0.573		
Sex				
Female	Reference		Reference	
Male	1.555 (0.925–2.756)	0.112		
Tumor size (cm)				
2–4	Reference		Reference	
≥4	0.778 (0.496–1.298)	0.331	1.756 (0.956–1.327)	0.024
Tumor location				
Head	Reference		Reference	
Body/Tail	0.956 (0.628–1.414)	0.753		
PS				
0	Reference		Reference	
1	0.678 (0.325–1.425)	0.687		
2	0.965 (0.456–2.105)	0.896		
CA 19-9 (U/ml)				
≤37	Reference		Reference	
>37	2.105 (1.015–4.356)	0.035	1.976 (0.945–3.975)	0.079
Preoperative therapy				
Hepaticojejunostomy	Reference		Reference	
Gastrojejunostomy	0.668 (0.279–1.582)	0.367		
Cholecystectomy	0.769 (0.285–1.689)	0.516		
Chemotherapy before IRE				
Gemcitabine	Reference		Reference	
S-1	0.685 (0.308–1.527)	0.355		
FOLFIRINOX	0.691 (0.320–1.490)	0.345		
PD-L1 expression, *n* (%)				
Positive	Reference		Reference	
Negative	0.689 (0.318–1.489)	0.352		
Unknown	0.691 (0.252–1.614)	0.381		
PD-1/L1 blockade, *n* (%)				
Camrelizumab	Reference		Reference	
Toripalimab	0.965 (0.725–1.268)	0.811		
Nivolumab	1.014 (0.774–1.337)	0.918		
Pembrolizumab	0.477 (0.278–1.082)	0.085		
Atezolizumab	0.676 (0.267–1.590)	0.381		
Treatment				
IRE+chemotherapy	Reference		Reference	
IRE+chemotherapy+PD-1/PD-L1 blockade	0.446 (0.239–0.780)	0.005	0.459 (0.248–0.824)	0.006

IRE, irreversible electroporation; PFS, progression-free survival; PD-1, programmed cell death protein 1; PD-L1, programmed death-ligand 1; HR, hazard ratio; CI, confidence interval; PS, performance status; CA19-9, carbohydrate antigen 19-9.

## Discussion

This study marks the first report on the antitumor immunity resulting from the combination of IRE, chemotherapy, and PD-1/PD-L1 blockade in patients with LAPC. Our findings suggest that the antitumor immunity effectiveness and survival advantage of this triplet therapy are notably superior to those of the doublet therapy. Furthermore, our multivariate analysis showed a significant association between triplet therapy and OS and PFS in patients with LAPC.

Although IRE has been established to stimulate tumor-specific T-cell immune responses (24, 30), the resulting antitumor immunity is typically insufficient to eliminate distant micrometastatic lesions. Therefore, integrating IRE with immunotherapy could potentially offer a promising strategy to address this concern. To date, only two studies have documented the application of IRE in combination with ICIs for LAPC (12, 31). In a phase 1b clinical trial, IRE followed by nivolumab was administered to 10 patients with stage III pancreatic cancer, with median OS and PFS times of 18.0 months significantly lower than the 23.6 months observed in our study (12). In another retrospective study, He et al. (31) compared the therapeutic impact of IRE plus toripalimab versus IRE alone for LAPC, revealing a median OS of 44.3 months, markedly higher than in our study. This difference can be attributed to two main factors. Firstly, all patients in their study underwent four months of induction chemotherapy, likely possessing a more stable physical condition than our cohort. Secondly, we found that patients in our study had a more significant burden of tumors.

Our study possesses two notable distinctions when contrasted with previous studies. Firstly, our study show cases of methodological robustness as it involves patients treated with a combination of irreversible electroporation (IRE), chemotherapy, and PD-1/PD-L1 blockade. While IRE has been substantiated to stimulate tumor-specific T-cell immune responses (24, 30), the resulting antitumor immunity is generally insufficient to eradicate distant micrometastatic lesions. Furthermore, IRE not only boosts cell membrane permeability, facilitating greater entry of chemotherapeutic drugs into tumor cells and enhancing their cytotoxicity, but it also attracts a more significant number of CD8^+^ T cells to invade tumor regions and activate the immune system (32). Secondly, unlike prior studies, all our patients did not undergo induction chemotherapy, and their tumor burdens were more considerable. Our findings demonstrated an increase in the frequency of lymphocytes and cytokines post-IRE treatment. These results suggest that IRE is capable of inducing an immune response, providing a therapeutic possibility for the combined use of PD-1/PD-L1 blockade in the treatment of LAPC patients.

In our research, the multivariate analysis determined that the combination of IRE, chemotherapy, and PD-1/PD-L1 blockade, as well as tumor size, could independently improve OS. Consistent with our findings, a study by He et al. demonstrated that tumor size, CA 125 levels, and the treatment strategy were associated with poor OS outcomes (33). Our results corroborate this observation, indicating that integrating PD-1/PD-L1 therapy could potentially offer enhanced clinical benefits for patients with LAPC.

Regarding safety, common adverse events related to IRE treatment include pain, fatigue, diarrhea, cardiac arrhythmias, and hypertension, which align with previous studies (34, 35). The characteristics of immune-related adverse events observed in our study were akin to those documented in prior research (33). All these adverse events could be mitigated with symptomatic treatment. In our research, seven patients (28%) reported grade 3 or higher adverse events, which is a lower percentage than in previous studies (70%) (12). This difference may be attributed to the potential synergistic effect of combination therapy, with the ultimate goal being a sustainable and synergistic therapeutic response.

Despite the promising results highlighted in this study, several limitations must be acknowledged. Firstly, as this is a single-center retrospective study, bias is inevitable. Secondly, the study’s small sample size necessitates the need for future multi-center, prospective, randomized controlled, and large-sample clinical studies. Lastly, the expression of PD-L1 serves as a key biomarker for PD-1/PD-L1 blockade therapy. However, these data could not be obtained for most patients, which might have indirectly influenced the results of our study.

In conclusion, the combination of IRE with chemotherapy and PD-1/PD-L1 blockade therapy could potentiate robust antitumor immunity. This three-pronged approach was independently linked to both OS and PFS for patients with LAPC.

## Data availability statement

The original contributions presented in the study are included in the article/supplementary material. Further inquiries can be directed to the corresponding author.

## Ethics statement

The studies involving humans were approved by Institutional Ethic Committee of Fuda Cancer Hospital. The studies were conducted in accordance with the local legislation and institutional requirements. The participants provided their written informed consent to participate in this study. Written informed consent was obtained from the individual(s) for the publication of any potentially identifiable images or data included in this article.

## Author contributions

LN designed the project and edited the manuscript. YM, YX, HL, TY, BL, RL, JL, ZL, and SL conducted study selection, data extraction, statistical analyses, and drafted the main manuscript. All authors contributed to the article and approved the submitted version.
